# PD-1/PD-L1 Inhibitors as Monotherapy in the First-Line Treatment of Advanced Non-Small Cell Lung Cancer Patients with High PD-L1 Expression: An Expert Position Statement

**DOI:** 10.3390/jcm12155063

**Published:** 2023-08-01

**Authors:** Dolores Isla, Alfredo Sánchez, Joaquín Casal, Manuel Cobo, Margarita Majem, Noemi Reguart, Jon Zugazagoitia, Reyes Bernabé

**Affiliations:** 1 Medical Oncology Department, University Hospital Lozano Blesa, 50009 Zaragoza, Spain; 2 Medical Oncology Department, Consorci Hospitalari Provincial de Castelló, 12002 Castelló, Spain; 3 Medical Oncology Department, Hospital Álvaro Cunqueiro, Área Sanitaria de Vigo, 36213 Vigo, Spain; 4 UGC Intercentros de Oncología Médica, Hospitales Universitarios Regional y Virgen de la Victoria, 29010 Málaga, Spain; 5 IBIMA, 29010 Málaga, Spain; 6 Medical Oncology Department, Hospital de la Santa Creu i Sant Pau, 08041 Barcelona, Spain; 7 Medical Oncology Department, Hospital Clínic, IDIBAPS, 08036 Barcelona, Spain; nreguart@clinic.cat; 8 Medical Oncology Department, 12 de Octubre University Hospital, 28041 Madrid, Spain; 9 Medical Oncology Department, Hospital Virgen del Rocio, Universidad de Sevilla, 41013 Sevilla, Spain

**Keywords:** lung cancer, non-small cell lung cancer, immunotherapy, first-line treatment, PD-(L)1 inhibitors, efficacy, safety, monotherapy, consensus

## Abstract

Introduction: There are currently three first-line immunotherapy options used as monotherapy in advanced non-small cell lung cancer (NSCLC) patients with high programmed death ligand 1 (PD-L1) expression (≥50%). This manuscript aims to evaluate the available data on atezolizumab (AT), cemiplimab (CEMI), and pembrolizumab (PEMBRO) and to study the results obtained during pivotal trials, especially regarding patient subgroups. Methods: Nominal group and Delphi techniques were used. Eight Spanish experts in lung cancer (the scientific committee of the project) analyzed the use of immunotherapy monotherapy as first-line treatment in patients with NSCLC and high PD-L1 expression. The expert scientific committee formulated several statements based on a scientific review and their own clinical experience. Subsequently, 17 additional Spanish lung cancer experts were selected to appraise the committee’s statements through two Delphi rounds. They completed a Delphi round via an online platform and voted according to a scale from 1 (strongly disagree) to 10 (strongly agree). The statements were approved if ≥70% of experts voted 7 or more. Results: A total of 20 statements were proposed covering the following areas: (1) general characteristics of pivotal clinical trials; (2) overall main outcomes of pivotal clinical trials; and (3) subgroup analysis. All statements reached consensus in the first round. Conclusions: AT, CEMI, and PEMBRO as monotherapy can be considered the standard of care in patients with advanced NSCLC and high PD-L1 expression (≥50%). Moreover, some differences noted among the drugs analyzed in this document might facilitate treatment decision-making, especially in clinically relevant patient subgroups, when using PD-1/PD-L1 inhibitors. The high level of agreement reached among experts supports the proposed statements.

## 1. Introduction

Lung cancer is the second-most common cancer and the deadliest type worldwide [1,2]. In 2020, the estimated age-standardized incidence rate in the United States (US) was 23.1 [2]. In the US, lung cancer is the third most frequent cancer but the leading cause of death, with approximately 127,070 deaths in 2023 [3], accounting for 18% of all cancer deaths [2].

Non-small cell lung cancer (NSCLC) represents about 80% of all lung cancers [3], and more than half of the new NSCLC cases are diagnosed with distant metastases [4]. Therefore, effective treatment in this setting is critical [5].

Immunotherapy targeting programmed cell death protein 1 (PD-1) and its ligand PD-L1 has significantly changed NSCLC patient management. PD-L1 is expressed by tumor cells (TCs) and tumor-infiltrating immune cells (ICs) [6]. PD-L1 binding to its receptor PD-1 on activated T-cells can lower T-cell immune responses and prevent TC elimination [7]. The prevalence of PD-L1 expression in the population of patients with NSCLC ranges from 24% to 60%, even with a cutoff for positivity set at 5% [8], and it is estimated that 25 to 35% of advanced NSCLC cases test positive for PD-L1 in at least 50% of TCs. Aside from being a key element in current immunotherapy strategies, PD-L1 has also emerged as a potential prognostic factor and biomarker to predict which patients are more likely to respond to immunotherapy in NSCLC [9]. 

PD-1/PD-L1 pathway inhibitors, either as monotherapy or combined with chemotherapy, with anti-CTLA4, and with or without bevacizumab, have markedly improved the overall survival (OS) and quality of life of NSCLC patients in different settings [10,11,12,13,14,15]. In pretreated NSCLC patients, PD-1/PD-L1 inhibitors led to significantly better overall responses, a longer OS, and reduced overall toxicity compared with chemotherapy [16]. Subgroup analysis according to tumor PD-L1 expression levels also showed that anti-PD-1/PD-L1 therapy significantly improved both OS and progression-free survival (PFS) in patients with high PD-L1 expression [16]. These results enabled the development of PD-1/PD-L1 inhibitors as first-line treatment in combination with chemotherapy, regardless of PD-L1 expression, and as monotherapy in PD-L1-positive patients [14]. Nowadays, two anti-PD-1 inhibitors, pembrolizumab (PEMBRO) [17], cemiplimab (CEMI) [18], and one anti-PD-L1 inhibitor, atezolizumab (AT) [19], have been approved as monotherapy in first-line treatment of adult NSCLC patients with high PD-L1 expression (in ≥50% tumor cells) with no EGFR, ALK, or ROS1 aberrations. 

Based on the published results, we built a national consensus statement with the aim of: (1) critically analyzing and describing the best evidence available of the efficacy and safety of PD-1/PD-L1 inhibitors as monotherapy in first-line treatment of advanced NSCLC; and (2) collecting evidence and experts’ opinions regarding the efficacy and safety of these drugs in several patient subgroups of clinical interest. We are confident that this document will be useful to improve NSCLC patient management. 

## 2. Methods

### 2.1. Study Design

A qualitative study was performed. Nominal group and Delphi techniques were applied to elaborate the consensus, with a systematic literature review supporting the statements. An external methodologist, expert in the Delphi technique, guaranteed the overall quality of the process. This study was conducted following good clinical practice and the current version of the revised Declaration of Helsinki (World Medical Association Declaration of Helsinki). 

### 2.2. Expert Panel Selection and Clinical Statement Generation

A scientific committee of eight experts on lung cancer was established as a first step. They were responsible for (1) selecting the expert panel involved in the Delphi process, (2) identifying current relevant clinical questions/controversies in the management of advanced NSCLC with PD-1/PD-L1 inhibitors, and (3) generating statements regarding the evidence and use of PD-1/PD-L1 inhibitors in clinical practice. These statements were organized into three main sections: (a) main characteristics of AT, CEMI, and PEMBRO pivotal trials; (b) main results of AT, CEMI, and PEMBRO pivotal trials; and (c) outcomes of AT, CEMI, and PEMBRO in predefined patient subgroups including age, sex, Eastern Cooperative Oncology Group (ECOG) performance status (PS) score, histological subtypes, presence of brain metastases, patients with unresectable locally advanced NSCLC, and PD-L1 levels. Along with the methodologists, the steering committee also defined the consensus level of agreement, and they analyzed and interpreted the results of the Delphi study. 

The expert panel invited to participate in the Delphi procedure comprised 25 experts that were selected according to the following criteria: they had to be (1) medical oncologists and (2) specialized in NSCLC with proven clinical expertise. In the selection process, a balanced territorial representation of Spain was considered. 

### 2.3. Systematic Literature Review

With the help of expert documentalists, a systematic literature review in Medline, Cochrane (CENTRAL), and Clinicaltrials.gov up to July 2022 was performed. The objective was to analyze the efficacy and safety of PD-1/PD-L1 inhibitors as monotherapy in first-line advanced NSCLC treatment. We used MeSH (Medical Subject Headings) terms (e.g., “carcinoma, non-small cell lung”) but also free text terms like “cemiplimab”. The inclusion criteria were: (1) adult patients with non-squamous or squamous NSCLC, measurable by response evaluation criteria in solid tumors (RECIST), version 1.1, and high PD-L1 expression (in ≥50% tumor cells); (2) treated with AT, CEMI, or PEMBRO as monotherapy in first-line treatment; (3) no restrictions were applied regarding the comparator; (4) articles should report outcomes like OS, PFS, objective response rate (ORR), or safety; (5) types of studies—meta-analysis, systematic literature reviews, and randomized controlled clinical trials—were accepted. Two reviewers independently selected the articles by reading the title and abstract, then thoroughly reading the whole article and collecting the data. The quality of the studies was analyzed using the Jadad score. Evidence and result tables were generated.

### 2.4. Delphi Process

The expert panel completed a Delphi round through an online platform. In the first round, the experts voted according to a scale from 1 (totally disagree) to 10 (totally agree). The statement was approved if more than 70% of participants voted ≥7. Statements with an agreement level <70% were analyzed, and if required, these statements were re-edited and voted on during a second round. 

### 2.5. Edition of the Document

The steering committee wrote the position statement document, supported by the narrative review results. 

## 3. Results

A total of 20 statements were generated. In the first Delphi round, all of them reached the required level of agreement. Delphi results are listed in Table 1. 

### 3.1. Main Characteristics of Pivotal Trials

**Figure 1:**
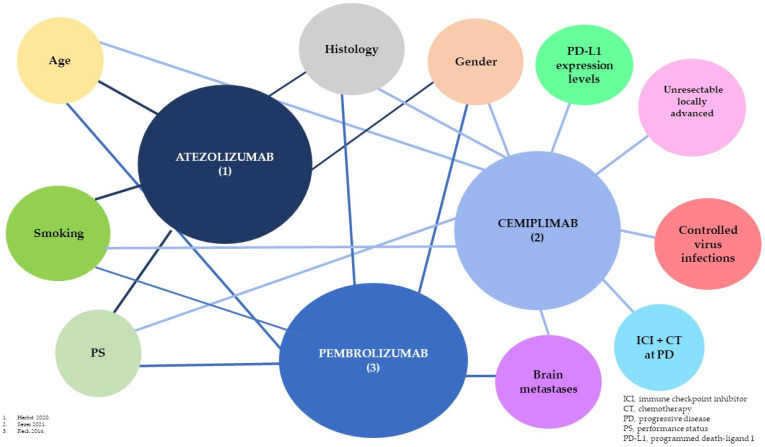
Atezolizumab, cemiplimab, and pembrolizumab trials with predefined designs and patientsubgroups [17,18,19].

Table 2, Table 3 and Table 4 and Figure 1 depict the main characteristics of AT, CEMI, and PEMBRO pivotal trials [17,18,19]. Corresponding statements are presented in Table 1 (statements 1 to 7), with a high grade of consensus observed. 

The AT, CEMI, and PEMBRO trials shared many characteristics but also presented several differences [17,18,19]. All were phase III randomized controlled trials in which the experimental drug was compared with platinum doublet chemotherapy (Table 2). However, in cases of disease progression, crossover was allowed in the CEMI and PEMBRO trials [17,18], but not in the AT trial [19]. In the CEMI trial, the continuation of immunotherapy after disease progression combined with chemotherapy was allowed [18]. 

Regarding inclusion and exclusion criteria, the three trials included patients with metastatic disease and a PS score of 0 or 1 [17,18,19]. In the CEMI study, locally advanced NSCLC patients who were not candidates for radical chemoradiotherapy were also allowed [18]. Smokers and former smokers were represented in all the trials, but the CEMI study did not include never smokers. Nevertheless, the CEMI trial considered patients with controlled hepatitis B virus (HVB), hepatitis C virus (HVC), and HIV infection [18]. Patients presenting baseline brain metastases (AT trial), untreated brain metastases (CEMI and PEMBRO trials), and those with any prior adjuvant or neoadjuvant treatment (AT and PEMBRO trials) were excluded. 

OS was the primary endpoint in the AT study [19], the PEMBRO study mainly focused on PFS [17], and the CEMI trial examined OS and PFS [18] (Table 2).

Baseline characteristics of the patients are shown in Table 3. The AT pivotal trial included 554 patients with a PD-L1 level >1%, of whom 205 had a PD-L1 expression ≥50% [19]. All patients in the CEMI and PEMBRO trials presented a PD-L1 expression ≥50% [18]. Globally, the pivotal trials analyzed patients with similar median ages [17,18,19]. The amount of male patients was slightly lower in the PEMBRO trial [17] (Table 3), while the rate of non-squamous cancers was higher [17] (slightly more than 80% in the control and treatment arms). The percentage of patients with brain metastases in the experimental arm was similar in the CEMI and PEMBRO trials [18]. Nevertheless, the number of patients with baseline brain metastases in the control arms was slightly lower in the PEMBRO trial than in the CEMI trial (6.6% vs. 12%) [18]. 

Based on these data, all the experts agreed that in NSCLC patients with high PD-L1 expression (≥50%) and PS score ≤ 1, first-line treatment with AT, CEMI, or PEMBRO should be considered (statement 7, Table 1). 

### 3.2. Main Results of Pivotal Trials 

Primary and secondary endpoint results of each pivotal trial are listed in Table 4 (see also Table 1, statements 8–13). Once again, it is important to highlight that a high grade of consensus was achieved by the expert panel.

First-line AT, CEMI, and PEMBRO as monotherapies were significantly superior to chemotherapy in NSCLC with PD-L1 expression ≥50% [18,20,21]. Treatment with AT, CEMI, and PEMBRO resulted in statistically longer OS and PFS than chemotherapy [18,20,21]. Objective response rates were also higher with the PD-L1 inhibitors than chemotherapy: 33.7% vs. 32.1% in the AT trial [21], 39% vs. 20% in the CEMI study [18], and 44.8% vs. 27.8% in the PEMBRO trial [17]. The CEMI and PEMBRO trials reported these results despite a high crossover rate [17,18]. Finally, AT, CEMI, and PEMBRO showed relevant improvements in health-related quality of life [18,22,23,24].

**Table 4:** Results of primary and secondary endpoints of atezolizumab, cemiplimab, and pembrolizumab trials.

	Atezolizumab *	Cemiplimab	Pembrolizumab
**Overall survival (months)**	- 20.2 vs. 14.7 - HR = 0.76 (95% CI [0.54, 1.09]) [21]	- NR vs. 14.2 - HR = 0.57 (95% CI [0.42, 0.77]) [18]	- 26.3 vs. 13.4 - HR = 0.62 (95% CI [0.48, 0.8]) [25]
**Progression-free survival (months)**	- 8.2 vs. 5.0 - HR = 0.59 (95% CI [0.43, 0.81]) [21]	- 8.2 vs. 5.7 - HR = 0.54 (95% CI [0.43, 0.68]) [18]	- 7.7 vs. 5.5 - HR = 0.50 (95% CI [0.39, 0.65]) [25]
**Objective response (months)**	- 40.2% vs. 28.6% - Median duration 38.9 vs. 8.3 [21]	- 39% vs. 20% - OR = 2.53 (95% CI [1.74, 3.69]) - Median duration 16.7 vs. 6.0 [18]	- 46.1% vs. 31.1% - Median duration 29.1 vs. 6.3 [25]
**Mean** **∆** **Global Health Status/HRQoL**	GHS = 62.8 vs. 59.9 [23]	∆ GHS/HRQoL15.9 vs. −8.3 [18]	QLQ-C30 = 6.95 (95% CI [3.29, 10.58]) vs. −0.88 (95% CI [−4.78, 3.02]) [24]

* Results refer to patients with PD-L1 expression in ≥50% of tumor cells or in ≥10% of tumor-infiltrating immune cells (high expression). **Abbreviations**: CI = confidence interval; GHS = Global Health Status; HR = hazard ratio; HRQoL = health-related quality of life; NR = not reached; OR = odds ratio; QLQ-C30 = Cancer Quality of Life Questionnaire.

Among all the patients evaluated for safety, treatment-related adverse events occurred in 60.5% of cases with AT [19], 57% with CEMI [18], and 87.2% with PEMBRO [25]. The rates of grade 3 and 4 adverse events were 30.1% and 28% for patients treated with AT and CEMI [18,19], respectively. Grade 3 and 4 treatment-related adverse events occurred in 15.4% of patients treated with PEMBRO [25] (no grade 5 events occurred in the PEMBRO trial). 

### 3.3. Main Results in Predefined Patient Subgroups in the Pivotal Trials

Several exploratory subgroup analyses have been reported with the use of first-line AT, CEMI, and PEMBRO monotherapies. A summary of these analyses is exposed in Table 5 and Table 6 (see also statements 14 to 20 of Table 1), and Figure 1. A consensus was reached by the expert panel, especially for statements 16 and 17.

In this setting, AT, CEMI, and PEMBRO have shown global efficacy, irrespective of age or sex [18,20,21,26] (Table 5). Interestingly, the clinical benefit obtained in men was significantly higher than chemotherapy in all pivotal trials and outcomes (OS, PFS) [18,20,21]. The respective HR for OS HR = 0.73 (95% CI [0.48, 1.11]) with AT [21], HR = 0.50 (95% CI [0.36, 0.69]) with CEMI [18], and HR = 0.54 (95% CI [0.36, 0.79]) with PEMBRO [20]. 

**Table 5:** Efficacy of first line atezolizumab, cemiplimab, and pembrolizumab monotherapies according to patient’s subgroups.

	Atezolizumab *	Cemiplimab	Pembrolizumab
**Overall survival**	**<65 years ** HR = 0.72 (95% CI [0.44, 1.19]) [21] **65–74 years ** HR = 0.78 (95% CI [0.45, 1.36]) [21] **>74 years** HR = 1.03 (95% CI [0.31, 3.48]) [21] --------------------------------------- **Men ** HR = 0.73 (95% CI [0.48, 1.11]) [21] **Women** HR = 0.84 (95% CI [0.45, 1.58]) [21] --------------------------------------- **ECOG PS score = 0** HR = 0.63 (95% CI [0.33, 1.20]) [21] **ECOG PS score = 1** HR = 0.80 (95% CI [0.53, 1.22]) [21] --------------------------------------- **Squamous** HR = 0.91 (95% CI [0.45, 1.83]) [21] **Non-squamous** HR = 0.72 (95% CI [0.48, 1.08]) [21]	**<65 years ** HR = 0.66 (95% CI [0.44, 1.00] [18] **≥65 years ** HR = 0.48 (95% CI [0.30, 0.76]) [18] --------------------------------------- **Men** HR = 0.50 (95% CI [0.36, 0.69]) [18] **Women** HR = 1.11 (95% CI [0.49, 2.52]) [18] --------------------------------------- **ECOG PS score = 0** HR = 0.77 (95% CI [0.41, 1.44]) [18] **ECOG PS score = 1** HR = 0.54 (95% CI [0.38, 0.76]) [18] --------------------------------------- **Squamous** HR = 0.48 (95% CI [0.30, 0.77]) [18] **Non-squamous** HR = 0.60 (95% CI [0.44, 0.83]) [18] --------------------------------------- **Brain metastases** HR = 0.42 (95% CI [0.20, 0.87]) [27] **No brain metastases** HR = 0.60 (95% CI [0.44, 0.83]) [18] --------------------------------------- **Locally advanced** HR = 0.67 (95% CI [0.38, 1.17]) [28]	**<65 years ** HR = 0.60 (95% CI [0.38, 0.96]) [20] **≥65 years** HR = 0.64 (95% CI [0.42, 0.98]) [20] **<75 years** HR = 0.71 (95% CI [0.59, 0.87]) [26] **≥75 years** HR = 0.41 (95% CI [0.23, 0.73]) [26] --------------------------------------- **Men** HR = 0.54 (95% CI [0.36, 0.79]) [20] **Women** HR = 0.95 (95% CI [0.56, 1.62]) [20] --------------------------------------- **ECOG PS score = 0** HR = 0.78 (95% CI [0.44, 1.37]) [20] **ECOG PS score = 1** HR = 0.56 (95% CI [0.39, 0.81]) [20] --------------------------------------- **Squamous** HR = 0.73 (95% CI [0.28, 1.39]) [20] **Non-squamous** HR = 0.58 (95% CI [0.41, 0.83]) [20] --------------------------------------- **Brain metastases** HR = 0.73 (95% CI [0.20, 2.62]) [20] **No brain metastases** HR = 0.64 (95% CI [0.46, 0.88]) [20]
**Progression-free survival**	-	**<65 years** HR = 0.51 (95% CI [0.37, 0.69]) [18] **≥65 years** HR = 0.60 (95% CI [0.43, 0.84]) [18] --------------------------------------- **Men** HR = 0.50 (95% CI [0.40, 0.64]) [18] **Women** HR = 0.79 (95% CI [0.43, 1.46]) [18] --------------------------------------- **ECOG PS score = 0** HR = 0.59 (95% CI [0.38, 0.92]) [18] **ECOG PS score = 1** HR = 0.52 (95% CI [0.41, 0.68]) [18] --------------------------------------- **Squamous** HR = 0.48 (95% CI [0.34, 0.67]) [18] **Non-squamous** HR = 0.60 (95% CI [0.44, 0.81]) [18] --------------------------------------- **Locally advanced** HR = 0.56 (95% CI [0.34, 0.95]) [28] --------------------------------------- **Brain metastases** HR = 0.34 (95% CI [0.18, 0.63]) [27]	-

* Results refer to patients with PD-L1 expression in ≥50% of tumor cells or in ≥10% of tumor-infiltrating immune cells (high expression). **Abbreviations**: HR = hazard ratio; CI = confidence interval; PS = performance status; HR = hazard ratio; ECOG = Eastern Cooperative Oncology Group.

Regarding PS score (Table 5), AT, CEMI, and PEMBRO demonstrated efficacy (or a tendency) in improving OS compared with chemotherapy in patients with PS scores of 0 or 1 [18,20,21]. CEMI also showed positive results when analyzing PFS in patients with PS 0 and 1 [18].

OS and PFS with CEMI were significantly higher than chemotherapy in both squamous (with very positive results) and non-squamous NSCLC patients [18]. OS was also significantly higher with AT (HR = 0.72, 95% CI [0.48, 1.308]) [21] and PEMBRO (HR = 0.58, 95% CI [0.41, 0.83]) [20] in non-squamous tumors. In squamous tumors, OS was numerically higher with PD-L1 inhibitors (Table 5). 

The CEMI and PEMBRO pivotal trials included patients with treated and clinically stable brain metastases at baseline [17,18]. OS was significantly higher with CEMI than with chemotherapy in patients with brain metastases (median follow-up 33.3 months, HR = 0.42, 95% CI [0.20, 0.87]) [27]. The ORR was also improved with CEMI (41.2%, 95% CI [24.6, 59.3]) versus chemotherapy (8.8%, 95% CI [1.9, 23.7]) [29]. However, although OS with PEMBRO was numerically superior to chemotherapy, it did not reach statistical significance [20] (Table 5). Considering all these data, the experts agreed that both CEMI and PEMBRO should be considered in patients with brain metastases, although CEMI results are more relevant. 

On the other hand, post hoc subgroup analysis in locally advanced NSCLC patients from the CEMI pivotal trial (Table 5) demonstrated improved OS and PFS with first-line CEMI compared with chemotherapy [28]. Therefore, the experts encourage clinicians to consider CEMI in unresectable, locally advanced NSCLC patients who are not candidates for radical chemoradiotherapy.

Finally, exploratory analysis of the CEMI pivotal trial showed that the magnitude of clinical benefits observed with CEMI was superior to chemotherapy and higher as PD-L1 expression increased (Table 6) [30]. This sub-analysis is currently not available for AT and PEMRBO. A consensus has been acquired considering these data (statement 20 of Table 1).

**Table 6:** Clinical benefits of first-line cemiplimab monotherapy by PD-L1 expression levels [18,30].

	PD-L1 ≥90%	PD-L1 >60% to <90%	PD-L1 ≥50% to ≤60%
**Overall survival (months)**	- NR vs. 13.3 - HR = 0.57 (95% CI [0.27, 1.10])	- NR vs. 14.2 - HR = 0.49 (95% CI [0.26, 0.92])	- NR vs. 11.7–PD-L1 - HR = 0.74 (95% CI [0.44, 1.24])
**Progression-free survival (months)**	- 12.7 vs. 6.1 - HR = 0.33 (95% CI [0.19, 0.58])	- 6.2 vs. 4.3 - HR = 0.57 (95% CI [0.38, 0.85])	- 4.3 vs. 6 - HR = 0.89 (95% CI [0.61, 1.29])
**ORR**	- 38.8% vs. 14.8%	- 39.5% vs. 16.7%	- 28% vs. 21.4%

**Abbreviations**: PD-L1 = programmed death-ligand 1; NR = Not reached; HR = hazard ratio; CI = confidence interval; ORR = objective response rate.

## 4. Discussion

Immunotherapy has emerged in recent years as a breakthrough therapy for NSCLC. The development of antibodies against PD-1 and its ligand, PD-L1, has dramatically transformed the therapeutic scenario for NSCLC patients. In advanced disease, the results of several randomized clinical trials have led to the approval of PD-L1 inhibitors in the first or subsequent treatment lines [31].

In this project, we have critically reviewed and analyzed the best evidence available regarding the efficacy and safety of PD-1/PD-L1 inhibitors as monotherapy in the first-line treatment of advanced NSCLC and in several patient subgroups of clinical interest. Based on the review, a set of related statements was proposed and voted on by an expert panel in a Delphi process. A high consensus grade was achieved by the expert panel in the first Delphi round as the required agreement level was reached in all the statements, thus reinforcing their value. This is the most relevant and noteworthy outcome of this project. Figure 1 summarizes the different patient profiles and the indicated treatments for them.

One of the main conclusions of our work is that, according to the experts and the reviewed literature, first-line PD-1/PD-L1 inhibitors as monotherapy can be considered the standard of care in advanced NSCLC patients with PD-L1 expression ≥50% without targetable mutations, in line with current clinical guidelines [5]. Significant benefits were observed regarding efficacy with PEMBRO [20] and CEMI [18], even when crossover was carried out, and benefits were particularly high with CEMI. We have also discussed the role of PD-L1 inhibitors as monotherapy in first-line treatment based on patient subgroups and their differences. 

It is important to note that in the CEMI trial [18], controlled HVB, HVC, and HIV infections were allowed, as were unresectable locally advanced NSCLC patients who were not candidates for radical chemoradiotherapy. 

Many lung cancer diagnoses occur in elderly patients, who are quite underrepresented in clinical trials. It is estimated that more than half of lung cancers are diagnosed in patients aged 65 or older [32]. Mortality is also higher with increasing age [32]. Therefore, this subgroup of patients deserves special consideration in clinical practice. Exploratory data from AT, CEMI, and PEMBRO pivotal trials have shown that elderly patients seem to obtain the same OS benefit as younger patients without additional toxicity [18,20,21,26]. Thus, age is not a limiting factor regarding AT, CEMI, and PEMBRO treatments.

Sex should also be considered since there are genetic, hormonal, and behavioral/lifestyle differences between males and females that might influence the response to immunotherapy [33,34]. We found that although AT, CEMI, and PEMBRO demonstrated efficacy regardless of sex [17,18,19,20,21,26], the clinical benefits (OS, PFS) obtained in men were more marked. Different meta-analyses have depicted quite similar results, suggesting sex-related differences in the response to immunotherapy [33]. The inherent strong immune response in females might explain, at least partly, why therapies enhancing the immune response are less effective in them compared to males. In contrast, the combination of immunotherapy and chemotherapy, or therapy enhancing the antigenicity of tumor cells, would be more effective in females than males [33]. However, the role of sex in cancer immunotherapy should be further explored. In the meantime, both male and female patients with advanced NSCLC are candidates for PD-1/PD-L1 inhibitors in first-line treatment as monotherapy.

ECOG PS score assessment is critical to oncologists treatment decision-making. In addition, the impact of the ECOG PS score on immunotherapy efficacy is already well known [35]. A recent metanalysis of real-world data showed that ECOG PS at treatment initiation represents a prognostic factor in patients with immunotherapy, with worse outcomes determined for those with poorer clinical conditions [36]. The three pivotal trials included patients with ECOG PS scores of 0 or 1. Since no differences were observed between the two groups, the experts stated that these drugs can be used independently of the ECOG PS score of 0 or 1 in clinical practice.

The other subgroup analyses were made according to histological types of cancer (squamous and non-squamous) in patients treated with AT, CEMI, and PEMBRO [18,19,20]. The frequency and efficacy results were higher for squamous cell carcinoma with CEMI. Overall, the results led the experts to consider the three immunotherapy options in both histologies. 

Brain metastases in lung cancer patients are very common. They are present in approximately 20 to 40% of cases and are potentially devastating complications in advanced lung cancer, leading to a decreased quality of life and an extremely poor prognosis [37,38]. Despite changes in NSCLC treatment options with the emergence of immunotherapy, there is still a degree of caution about using these new drugs for the treatment of lung cancer patients with brain metastases. In the past, an absent lymphatic system and blood-brain barrier were considered responsible for poor brain immunogenicity [39]. The CEMI and PEMBRO trials included patients with treated and clinically stable brain metastases, accounting for approximately 10% of the enrolled patients [17,18]. CEMI was significantly superior to chemotherapy in OS and ORR [18,27], while PEMBRO only demonstrated a positive tendency over chemotherapy regarding OS [17,20]. However, these results should be interpreted with caution due to the small sample size. The experts finally concluded that CEMI or PEMBRO should be considered in patients with brain metastases, but also noted that current data are more relevant for CEMI. 

Approximately one-third of NSCLC diagnoses are classified as locally advanced, resectable, or unresectable [40]. The CEMI trial included a notable proportion of unresectable locally advanced NSCLC patients who were not candidates for definitive chemoradiation (15% in the PD-L1 ≥50% population and 16% in the intention-to-treat population) [18]. In a post-hoc analysis, first line CEMI monotherapy demonstrated improved survival benefits compared with chemotherapy [28]. Considering these data, the expert panel supports the inclusion of unresectable, locally advanced NSCLC patients who are not candidates for radical chemoradiotherapy for CEMI treatment.

Finally, the effect of PD-L1 expression level on treatment response was assessed only in patients treated with CEMI. We have shown that the benefits (OS, PFS, and ORR) with CEMI [30] were superior to chemotherapy and incrementally associated with PD-L1 expression levels. Thus, baseline PD-L1 expression levels may be used to identify advanced NSCLC patients who will likely benefit the most from first-line treatment with CEMI.

One of the main limitations of this project was the lack of direct cross-comparison studies or comparisons between trials. In addition, several differences in the trial design might explain differences in the baseline characteristics of the included patients, limiting their comparability. For example, randomization stratification, previous chemotherapy, and smoking status. It is worth mentioning gender disparities across trials. The pivotal CEMI trial [18] had far fewer women (84 in both groups) than men (479 in both groups), compared to the PEMBRO trial [17] (118 women vs. 187 men in both groups) and the AT trial [19] (62 women vs. 143 men in both groups). More research is needed to clarify the impact of these differences in treatment response.

This is why we conducted this Delphi project: to obtain expert opinions to deliver statements trying to resolve uncertainties. In this regard, the strength of this study lies in the high level of agreement achieved among a broad group of expert oncologists. On the other hand, we included many subgroup analyses, which are still considered exploratory and require further confirmation. Nevertheless, they provide provisional and relevant data for treatment decision-making. In addition, some non-significant results are likely due to a lack of statistical power (low sample size, etc.). It is important to note that we performed a comprehensive literature review that was critically interpreted by a broad committee of clinical oncologists, providing a balanced view of the diseases and treatments.

In summary, PD-1/PD-L1 inhibitors as first-line monotherapy can be considered the standard of care in patients with advanced NSCLC and PD-L1 expression ≥50% without targetable mutations. We expect that this work will improve treatment decision-making, especially in clinically relevant patient subgroups displaying differences when treated with PD-1/PD-L1 inhibitors. The remarkably high agreement level reached among experts supports the proposed statements.

## Figures and Tables

**Table 1:** Delphi method results obtained from the three document sections.

#	Statement	n	% ≥7	Mean (SD)	Median	p25–p75	Min	Max	Consensus
▪ Main characteristics of AT, CEMI, and PEMBRO pivotal clinical trials									
**1**	AT, CEMI, and PEMBRO have been compared with platinum-based chemotherapy in phase III studies whose primary endpoints are OS and PFS	25	96%	9.5 (6.3)	10	9–10	3	10	Consensus
**2**	Crossover was allowed in the CEMI and PEMBRO pivotal trials. The AT pivotal trial did not contemplate crossover	25	92%	9.2 (1.6)	10	9–10	4	10	Consensus
**3**	CEMI pivotal trial allowed the inclusion of patients with controlled hepatitis B virus, hepatitis C virus, and HIV infection and included only patients who were smokers or former smokers	25	96%	9.3 (1.4)	10	10–10	3	10	Consensus
**4**	AT pivotal trial included patients with PD-L1 expression ≥1%. CEMI and PEMBRO pivotal trials patients with PD-L1 expression ≥50%	24	96%	9.7 (1.1)	10	10–10	5	10	Consensus
**5**	CEMI pivotal trial included patients with unresectable locally advanced disease who were not candidates for radical chemoradiotherapy	25	100%	9.6 (0.2)	10	10–10	9	10	Consensus
**6**	CEMI pivotal trial allowed the continuation of immunotherapy after disease progression combined with chemotherapy	25	100%	9.8 (0.4)	10	10–10	8	10	Consensus
**7**	In advanced NSCLC patients with high PD-L1 expression (≥50%) and a PS score ≤ 1, first-line treatment with AT, CEMI, or PEMBRO should be considered	24	100%	9.8 (0.4)	10	10–10	8	10	Consensus
▪ Main results of AT, CEMI, and PEMBRO pivotal trials									
**8**	AT has been shown to be effective in OS and PFS with an acceptable safety profile	25	92%	8.7 (1.3)	10	8–10	6	10	Consensus
**9**	CEMI has been shown to be effective in OS and PFS with an acceptable safety profile	25	100%	8.9 (0.5)	10	10–10	8	10	Consensus
**10**	PEMBRO has been shown to be effective in OS and PFS with an acceptable safety profile	25	100%	9.8 (0.4)	10	10–10	9	10	Consensus
**11**	CEMI and PEMBRO pivotal trials had a high crossover rate	25	92%	9.1 (1.5)	9	8–10	4	10	Consensus
**12**	In CEMI, PEMBRO, and AT pivotal trials, a better response rate has been demonstrated compared with chemotherapy	25	100%	9.4 (0.9)	10	9–10	7	10	Consensus
**13**	CEMI, PEMBRO, and AT pivotal trials have shown an improvement in patients’ quality of life compared with chemotherapy	25	92%	9.1 (1.3)	10	9–10	5	10	Consensus
▪ Results of AT, CEMI, and PEMBRO in predefined patient subgroups									
**14**	The clinical benefits of AT, CEMI, and PEMBRO have been demonstrated irrespective of age (<65 years/≥65 years)	25	92%	8.8 (2.3)	9	8.25–10	2	10	Consensus
**15**	The clinical benefits of AT, CEMI, and PEMBRO have been observed irrespective of sex, with significant clinical benefits in male	25	88%	8.5 (2.3)	9	8–10	2	10	Consensus
**16**	The clinical benefits of AT, CEMI, and PEMBRO have been observed irrespective of a PS score of 0 or 1	25	100%	9.4 (0.7)	10	9–10	8	10	Consensus
**17**	In patients with squamous and non-squamous histological subtypes, the use of immunotherapy should be considered	25	100%	9.8 (0.5)	10	10–10	8	10	Consensus
**18**	Treatment with CEMI or PEMBRO should be considered in patients with brain metastases, with more relevant results for CEMI. No data are currently available for AT	25	76%	8 (1.9)	9	7–10	4	10	Consensus
**19**	CEMI should be considered in unresectable, locally advanced NSCLC patients who are not candidates for radical chemoradiotherapy	25	88%	8.7 (1.5)	9	8–10	5	10	Consensus
**20**	The magnitude of the clinical benefit observed with CEMI were incrementally associated with PD-L1 expression levels. There are no corresponding data for AT and PEMBRO	25	88%	8.3 (2.1)	9	8–10	1	10	Consensus

**Abbreviations:** AT = atezolizumab; CEMI = cemiplimab; PEMBRO = pembrolizumab; OS = overall survival; PFS = progression-free survival; PS = performance status; NSCLC = non-small cell lung cancer; PD-L1 = programmed death ligand 1; HIV = human immunodeficiency virus; min = minimum; max = maximum; SD = standard deviation.

**Table 2:** Atezolizumab, cemiplimab, and pembrolizumab pivotal trial designs and treatment schemes.

	Atezolizumab [19] IMpower110- NCT02409342	Cemiplimab [18] EMPOWER-Lung 1, NCT03088540	Pembrolizumab [17] KEYNOTE-024 NCT02142738
**Phase III open**	✓	✓	✓
**Randomization 1:1**	✓	✓	✓
**Randomization stratification**	- Sex - Performance Status - Histology - PD-L1 status	- Histology - Geographical region	- Performance status - Histology - Geographical region
**Previous chemotherapy**	- No	- Adjuvant (2.9%) - Neoadjuvant (1.3%)	- No
**Experimental arm**	- 1200 mg iv/3 weeks	- 350 mg iv/3 weeks	- 200 mg iv/3 weeks
**Control arm**	- Squamous NSCLC: cisplatin or carboplatin + pemetrexed - Non-squamous NSCLC: cisplatin + gemcitabine or regimen of carboplatin + gemcitabine	- Pemetrexed + cisplatin - Pemetrexed + carboplatin - Paclitaxel + cisplatin - Paclitaxel + carboplatin - Gemcitabine + cisplatin - Gemcitabine + carboplatin	- Carboplatin + pemetrexed - Cisplatin + pemetrexed - Carboplatin + gemcitabine - Cisplatin + gemcitabine - Carboplatin + paclitaxel
**If disease progression**	- Experimental arm: switch to chemotherapy (continuation of AT allowed) - Control arm: no switch to AT allowed	- Experimental arm: switch to chemotherapy (continuation of CEMI allowed) - Control arm: switch to CEMI	- Experimental arm: chemotherapy treatment plan not pre-established - Control arm: switch to PEMBRO
**Primary endpoint**	- OS	- OS - PFS	- PFS
**Secondary endpoint**	- PFS - Objective response occurrence and duration	- ORR - Response duration	- OS - Objective response occurrence and duration

**Abbreviations**: mg = milligram; iv = intravenous; OS = overall survival; PFS = progression-free survival; PD-L1 = programmed death ligand 1; NSCLC = non-small cell lung cancer; AT = atezolizumab; CEMI = cemiplimab; PEMBRO = pembrolizumab; ORR = objective response rate.

**Table 3:** Baseline main characteristics of patients of atezolizumab, cemiplimab, and pembrolizumab pivotal trials.

	Atezolizumab [19] IMpower110- NCT02409342		Cemiplimab [18] EMPOWER-Lung 1, NCT03088540		Pembrolizumab [17] KEYNOTE-024 NCT02142738	
n Experimental arm Control arm	107 * 98 *		283 ^†^ 280		154 151	
Median age ^‡^ Experimental arm Control arm	63 (33–79) 66 (33–87)		63 (58–69) 64 (58–70)		64 (33–90) 66 (38–85)	
Male patients n (%) Experimental arm Control arm	79 (73.8) 64 (65.3)		248 (88) 231 (83)		92 (59.7) 95 (62.9)	
ECOG PS n (%) Experimental arm Control arm	0 35 (32.7) 38 (38.8)	1 72 (67.3) 60 (61.2)	0 77 (27) 75 (27)	1 206 (73) 205 (73)	0 54 (35.1) 53 (35.1)	1 99 (64.3) 98 (64.9)
Histology n (%) Experimental arm Control arm	Squamous 27 (25.2) 23(23.5)	Non-squamous 80 (74.8) 75 (76.5)	Squamous 122 (43) 121 (43)	Non-squamous 161 (57) 159 (57)	Squamous 29 (18.8) 27 (17.9)	Non-squamous 125 (81.2) 124 (82.1)
Brain metastases n (%) Experimental arm Control arm	- -		34 (12) 34 (12)		18 (11.7) 10 (6.6)	

* Patients with PD-L1 expression in ≥50% of tumor cells or in ≥10% of tumor-infiltrating immune cells (high expression). ^†^ PD-L1≥50% population (not the ITT population of EMPOWER-Lung1. ^‡^ (p25–p75). **Abbreviations**: ECOG = Eastern Cooperative Oncology Group; PS = Performance status.
